# Editorial: Mammalian spermatogenesis: genetic and environmental factors

**DOI:** 10.3389/fcell.2024.1372799

**Published:** 2024-01-31

**Authors:** Wei Qu, Xinnai Yu, Hanqing Shi, Zhiyi Chen, Mengcheng Luo

**Affiliations:** Provincial Key Laboratory of Developmentally Originated Disease, TaiKang Center for Life and Medical Sciences, School of Basic Medical Sciences, Wuhan University, Wuhan, Hubei, China

**Keywords:** spermatogenesis, genetic, environment, spermatogonia stem cell, meiosis, sperm

Spermatogenesis is a complex and tightly regulated process, which includes the proliferation of spermatogonia, spermatogonia differentiation into spermatocytes, meiotic division of spermatocytes producing spermatids, maturation of round spermatids and the release of highly specialized mature spermatozoa (Neto et al., 2016). Abnormalities in any of these events could lead to spermatogenesis disorders that affect fertility. Spermatogenesis disorders can be caused by genetic and non-genetic factors, of which genetic accounts for 15%–30% and non-genetic accounts for 70%–85% (O'Flynn O'Brien et al., 2010; Neto et al., 2016). Notably, environmental factors, as one of the non-genetics, are important for spermatogenesis because the male reproductive system, especially spermatogenesis, seems to be particularly sensitive to environmental hazards (Vecoli et al., 2016). This Research Topic includes seven original articles and one mini review that enhance and expand our knowledge regarding these factors and mechanisms.

Spermatogonia stem cells (SSCs) are the most primitive germ cells, which maintain spermatogenesis by self-renewal and continuous differentiation into spermatocytes to produce spermatozoa in the testis (Kubota and Brinster, 2018). The study by Wu et al. found that GPx3 regulates the proliferation and apoptosis of human SSCs. The authors showed that GPx3 is highly expressed in human SSCs, and its knockdown inhibits cell proliferation. Besides, GPx3 interacts with CXCL10, and their knockdown phenotypes are consistent in the human SSC line. The results illustrate that GPx3 and CXCL10 are essential for SSC self-renewal. There are a few studies about the influence of external environmental factors on the self-renewal and differentiation of SSCs. Previous studies have shown that hypoxia is beneficial for the proliferation of SSCs (Morimoto et al., 2021). In this Research Topic, Gille et al. investigated how hypoxia affects SSC proliferation and differentiation. The authors demonstrated that SSCs show a slight differentiation bias and a decrease in proliferation when O2 tension ≤1%, which is consistent with the results of Morimoto et al. (2021).

There are several important events that occur during meiosis, including DNA replication, chromatin condensation, DSB formation, and DSB repair. These events are not exclusive to meiosis and occur in the cycle of somatic cells, and nuclear actin has been shown to be associated with these events. However, there are no studies to elucidate the relationship between nuclear actin and meiosis. In this Research Topic, Petrusová et al. provide a mini review to elucidate the functions of nuclear actin in prophase I spermatocytes. The authors propose that nuclear actin may recruit INO80 to DNA loci to activate transcription and perform DNA break repair in meiosis. Since the NuA4 complex, SWI/SNF complex and NuRD complex are associated with DNA repair, chromatin structure and synapsis, actin may have played a role as one of the components of these complexes.

The blood-testis barrier is one of the blood-tissue barriers in mammals, providing a unique microenvironment for the completion of meiosis and subsequent development of spermatids into spermatozoa (Mruk and Cheng, 2015). The presence of a blood-testis barrier prevents damage to germ cells caused by external environment factors, including chemicals, microplastics, and so on (Zhou et al., 2022). Venditti et al. confirmed that microplastics and cadmium can penetrate the blood-testis barrier and disrupt the process of spermatozoa differentiation into spermatozoa in mice. They further identified the decreased expression of PTMA, a small peptide that regulates germ cell proliferation and differentiation, and DAAM1 and PREP, two proteins involved in actin- and microtubule-associated processes during germ cell differentiation into spermatozoa. Furthermore, Zhang et al. found that the inhalation of anesthetic sevoflurane inhibited spermatogenesis by reducing oxidative phosphorylation through inducing iron deficiency. Sevoflurane disrupts the blood-testicular barrier, inhibits germ cell proliferation, and promotes apoptosis in mouse testis. Interestingly, iron supplementation increases sperm concentration and inhibits the damage of sevoflurane to the testis.

During sperm maturation, 90%–95% of the histones are replaced by protamine, and retained histones contain epigenetic modifications that are responsible for the activation and silencing of paternal genes after fertilization (Carrell, 2012). The deposition and removal of these epigenetic modifications are the responsibility of epigenetic enzymes. Barbero et al. mapped the expression patterns of epigenetic enzymes in germ cells differentiating into the mature sperm. From published transcriptome data, the authors found that the transition of the spermatogenesis stage is characterized by writing erasure of histone K methylation and acetylation. Epigenetic enzymes act as vectors of epigenetic information transmission by participating in the maternal-to-zygote transition.

The sperm with motility loss are unable to reach the oocyte, which is the cause of male infertility due to severe asthenozoospermia (Freitas et al., 2017). The sperm flagella are necessary for the motility and integrity of sperm, and it possess a “9 + 2” structure, in which a central pair of singlet microtubules is cylindrically surrounded by nine microtubule doublets (Liu et al., 2023). Meng et al. described that the novel homozygous variants in TTC12 cause asthenozoospermia. The authors identified 3 patients with TTC12 mutation out of 314 asthenoteratozoospermia-affected men, and the mutations result in morphological abnormalities of flagella, with the absence of outer and inner dynein arms. Notably, 3 patients with TTC12 mutation have healthy offspring after sperm is treated with intracytoplasmic sperm injection technology. In addition to genetic factors, the immaturity of semen cryopreservation technology also affects sperm motility. Despite continuous optimization of sperm cryopreservation procedures and reduction of cryoprotectant toxicity, sperm viability and motility are inevitably decreased, which seriously affects fertilization ability (Whaley et al., 2021). It has been reported that numerous genes are correlated with sperm fertility and freezing tolerance. For example, the SNPs in bovine FSHβ are associated with semen quality and fertility in bulls (Khan et al., 2021). Bai et al. integrated transcriptomics and proteomics approaches and screened out FCGR1A as an important factor in preserving sperm fertilization capacity during semen cryopreservation in sheep. After FCGR1A is blocked, the sperm viability and cleavage rate of embryos decrease significantly.

We are grateful to all contributors for their excellent research on this Research Topic and hope that this Research Topic will provide new insights into mammalian spermatogenesis and advance the field.
